# Associations Between Frailty and the Increased Risk of Adverse Outcomes Among 38,950 UK Biobank Participants With Prediabetes: Prospective Cohort Study

**DOI:** 10.2196/45502

**Published:** 2023-05-18

**Authors:** Xingqi Cao, Xueqin Li, Jingyun Zhang, Xiaoyi Sun, Gan Yang, Yining Zhao, Shujuan Li, Emiel O Hoogendijk, Xiaofeng Wang, Yimin Zhu, Heather Allore, Thomas M Gill, Zuyun Liu

**Affiliations:** 1 Center for Clinical Big Data and Analytics of the Second Affiliated Hospital Zhejiang University School of Medicine Hangzhou China; 2 Department of Big Data in Health Science School of Public Health Zhejiang University School of Medicine Hangzhou China; 3 The Key Laboratory of Intelligent Preventive Medicine of Zhejiang Province Zhejiang University School of Medicine Hangzhou China; 4 Department of Neurology, Fuwai Hospital National Center for Cardiovascular Diseases Peking Union Medical College, Chinese Academy of Medical Sciences Beijing China; 5 Department of Epidemiology & Data Science Amsterdam Public Health Research Institute Amsterdam University Medical Center Amsterdam Netherlands; 6 National Clinical Research Center for Aging and Medicine Huashan Hospital Shanghai China; 7 Human Phenome Institute Fudan University Shanghai China; 8 Department of Epidemiology & Biostatistics School of Public Health Zhejiang University School of Medicine Hangzhou China; 9 Department of Internal Medicine Yale School of Medicine New Haven, CT United States

**Keywords:** frailty, adverse outcomes, diabetes, prediabetes, prospective study

## Abstract

**Background:**

Compared with adults with normal glucose metabolism, those with prediabetes tend to be frail. However, it remains poorly understood whether frailty could identify adults who are most at risk of adverse outcomes related to prediabetes.

**Objective:**

We aimed to systematically evaluate the associations between frailty, a simple health indicator, and risks of multiple adverse outcomes including incident type 2 diabetes mellitus (T2DM), diabetes-related microvascular disease, cardiovascular disease (CVD), chronic kidney disease (CKD), eye disease, dementia, depression, and all-cause mortality in late life among middle-aged adults with prediabetes.

**Methods:**

We evaluated 38,950 adults aged 40 years to 64 years with prediabetes using the baseline survey from the UK Biobank. Frailty was assessed using the frailty phenotype (FP; range 0-5), and participants were grouped into nonfrail (FP=0), prefrail (1≤FP≤2), and frail (FP≥3). Multiple adverse outcomes (ie, T2DM, diabetes-related microvascular disease, CVD, CKD, eye disease, dementia, depression, and all-cause mortality) were ascertained during a median follow-up of 12 years. Cox proportional hazards regression models were used to estimate the associations. Several sensitivity analyses were performed to test the robustness of the results.

**Results:**

At baseline, 49.1% (19,122/38,950) and 5.9% (2289/38,950) of adults with prediabetes were identified as prefrail and frail, respectively. Both prefrailty and frailty were associated with higher risks of multiple adverse outcomes in adults with prediabetes (*P* for trend <.001). For instance, compared with their nonfrail counterparts, frail participants with prediabetes had a significantly higher risk (*P*<.001) of T2DM (hazard ratio [HR]=1.73, 95% CI 1.55-1.92), diabetes-related microvascular disease (HR=1.89, 95% CI 1.64-2.18), CVD (HR=1.66, 95% CI 1.44-1.91), CKD (HR=1.76, 95% CI 1.45-2.13), eye disease (HR=1.31, 95% CI 1.14-1.51), dementia (HR=2.03, 95% CI 1.33-3.09), depression (HR=3.01, 95% CI 2.47-3.67), and all-cause mortality (HR=1.81, 95% CI 1.51-2.16) in the multivariable-adjusted models. Furthermore, with each 1-point increase in FP score, the risk of these adverse outcomes increased by 10% to 42%. Robust results were generally observed in sensitivity analyses.

**Conclusions:**

In UK Biobank participants with prediabetes, both prefrailty and frailty are significantly associated with higher risks of multiple adverse outcomes, including T2DM, diabetes-related diseases, and all-cause mortality. Our findings suggest that frailty assessment should be incorporated into routine care for middle-aged adults with prediabetes, to improve the allocation of health care resources and reduce diabetes-related burden.

## Introduction

In 2021, the International Diabetes Federation estimated that there were more than 500 million adults with prediabetes among those aged 20 years to 79 years worldwide [1]. As an intermediate hyperglycemia state, prediabetes increases the risk of diabetes [2] and diabetes-related complications (eg, cardiovascular disease [CVD], chronic kidney disease [CKD], and dementia) [3]; the latter contributes to a large proportion of diabetes-related burden [4,5]. The latest guidelines from the American Diabetes Association (ADA) recommend annual diabetes screening for adults with prediabetes [6]. However, this is challenged by emerging evidence showing the very low rates of diabetes progression among older adults with prediabetes [7]. Conversely, middle-aged adults (ie, <65 years) with prediabetes should be monitored for adverse outcomes, which is of high value and appropriate [8].

Prediabetes is highly heterogeneous, impeding the application of a one-size-fits-all health management strategy. Recently, a simple health aging indicator—frailty—has been demonstrated to be able to predict the risk of adverse outcomes (eg, CVD and mortality) [9-12] even in the younger population [13]. Frailty is defined as a state of decreased reserve and resistance to stressors, characterized by functional decline in multiple systems [9]. Frailty and disorders of glucose metabolism share common physiological mechanisms, such as insulin resistance [14,15] and chronic inflammation [15,16]. Frailty has been found to be an important risk factor for disability [17], fracture [18], CVD [19,20], hospitalization [20], intensive care unit admission [20], and mortality [20,21] among adults with diabetes. A few studies have shown that frailty incidence is slightly higher in older adults with prediabetes compared with those with normal glucose metabolism [22]. Only 1 prospective study recently reported that frailty was positively associated with the progression of prediabetes to type 2 diabetes mellitus (T2DM), as well as higher risks of CVD and all-cause mortality, in middle-aged and older adults with prediabetes [23]. However, whether these positive associations remain in those aged less than 65 years is not yet clear. In addition, impaired glucose metabolism is also associated with higher risks of CKD [3], eye disease (eg, cataract) [24], dementia [3], and depression [25]. However, relatively little is known about whether frailty could identify middle-aged adults with prediabetes who are most at risk of these adverse outcomes.

Therefore, we performed a prospective cohort study among 38,950 middle-aged adults with prediabetes from the UK Biobank (UKB). Using a widely validated frailty measurement—frailty phenotype (FP) [9]—the objective of this study was to systematically evaluate the associations of frailty with the risk of multiple adverse outcomes, including incident T2DM, diabetes-related microvascular disease, CVD, CKD, eye disease, dementia, depression, and all-cause mortality.

## Methods

### Study Participants

**Figure 1:**
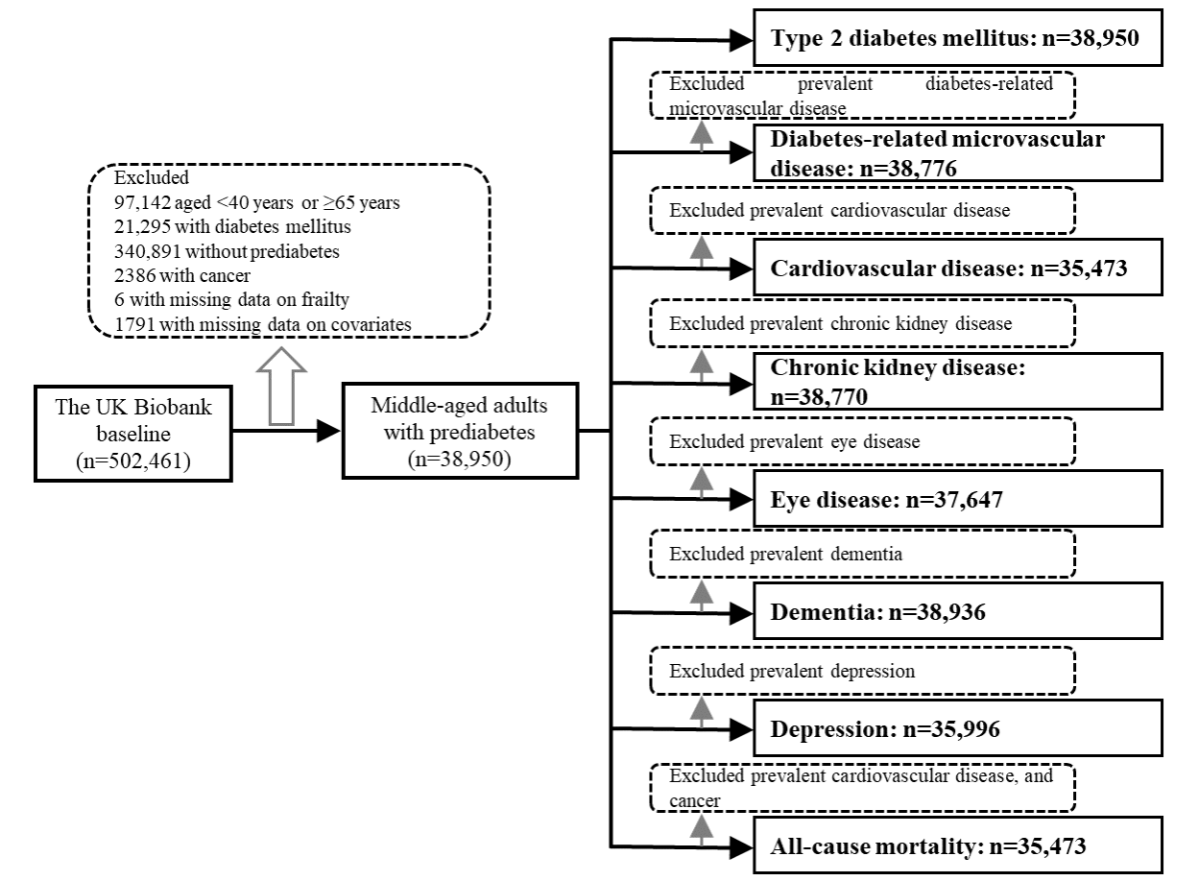
Flow chart of the sample for analyses.

The UKB is a large-scale health research study with a long-term follow-up that began in 2006 to 2010 with the recruitment of approximately 500,000 adults in the United Kingdom [26]. Adults in the UKB were recruited through 22 assessment centers across England, Scotland, and Wales. Data were collected through a touch screen questionnaire and verbal interviews (eg, demographic, health, lifestyle variables), physical measures (eg, handgrip strength), and biological sample collection (eg, blood). Since recruitment, all adults have given consent for the UKB to follow up to determine the incidence of health outcomes through links to health-related records (eg, hospital inpatient episodes and death registrations), and only about 0.3% of the adults have been lost to follow-up because they left the United Kingdom or withdrew consent for future linkage. The protocol of the UKB is available online [27]. At baseline, there were 405,319 middle-aged adults (age: 40-64 years), of whom 43,133 had prediabetes. Prediabetes was defined by a nonfasting glycated hemoglobin (HbA_1c_) level of 5.7% to 6.4% (39-47 mmol/mol) following the ADA criteria [6]. After the exclusion of adults with prevalent cancer (n=2386) or with missing data on frailty (n=6) and covariates (eg, ethnicity, educational level; n=1791), 38,950 middle-aged adults with prediabetes were included in the final analytic samples. Additionally, because the number of prevalent cases for each outcome varied, we assembled different analytic samples for each outcome (see details in Figure 1).

### Ethical Considerations

The UKB was approved by the North West Multi-Centre Research Ethics Committee (11/NW/0382). Written informed consent from all participants was obtained. The data used in this study were anonymized and de-identified for privacy and confidentiality protection.

### Outcomes

In this study, the outcomes included T2DM, diabetes-related microvascular disease (including retinopathy, neuropathy, and nephropathy), diabetes-related macrovascular disease (ie, CVD including ischemic heart disease and stroke), CKD, eye disease (including cataract and glaucoma), dementia, depression, and all-cause mortality.

We defined prevalent and incident T2DM using a UKB algorithm that combined self-reported medical history and medication information (for the ascertainment of prevalent cases only), as well as linked hospital admissions records (Table S1 in Multimedia Appendix 1). In addition, according to the ADA criteria [6], undiagnosed prevalent T2DM cases were identified using random glucose (≥11.1 mmol/L) or HbA_1c_ (≥6.5% [48 mmol/mol]) levels. We ascertained prevalent and incident cases of diabetes-related microvascular disease through linked hospital admissions records using the International Statistical Classification of Diseases and Related Health Problems, 9th version (ICD-9) and 10th version (ICD-10; Table S1 in Multimedia Appendix 1). We ascertained prevalent and incident cases of CVD, CKD, eye disease, dementia, and depression using self-reported medical history (for the ascertainment of prevalent cases only) and linked hospital admissions records using ICD-9 and ICD-10 (Table S1 in Multimedia Appendix 1). We ascertained death through linkage to national death registries. For analyses of each outcome, the time to event was calculated from the baseline (ie, the years 2006-2010) to the occurrence of the specific disease outcome, death, loss to follow-up, or end of follow-up (the year 2021), whichever came first. For instance, for analysis of incident T2DM, time to event was calculated from the baseline to the occurrence of T2DM, death, loss to follow-up, or end of follow-up, whichever came first.

### Frailty Measurement

We used FP, a widely used physical frailty measurement proposed by Fried et al [9]. FP was evaluated using 5 criteria (unintentional weight loss, exhaustion, weakness, slow gait speed, and low physical activity) and was used previously in the UKB [28]. Of the 5 criteria, weakness was assessed using objectively measured handgrip strength; the other 4 criteria were assessed using a self-reported questionnaire (see details in Table 1). The FP score ranged from 0 to 5, with a higher score indicating greater frailty. Participants were categorized into nonfrail (FP score=0), prefrail (FP score≥1 and ≤2), and frail (FP score≥3), as done in previous studies [9,28].

**Table 1:** The 5 criteria for the frailty phenotype in the UK Biobank.

Number	Criteria description	Categories
1	Unintentional weight loss: Participants were asked “Compared with one year ago, has your weight changed?”	1: “Yes, loss weight”; 0: Others
2	Exhaustion: Participants were asked “Over the past 2 weeks, how often have you felt tired or had little energy?”	1: “More than half the days or nearly every day”; 0: Others
3	Weakness: Weakness was measured using grip strength with a Jamar J00105 hydraulic hand dynamometer (Lafayette Instrument). Participants were asked to complete a grip assessment for both hands once. The maximal value of the right and left hands was used.	1: (1) Men: ≤29 kg for BMI ≤24 kg/m^2^; ≤30 kg for BMI 24.1-26 kg/m^2^; ≤30 kg for BMI 26.1-28 kg/m^2^; or ≤32 kg for BMI >28 kg/m^2^; (2) Women: ≤17 kg for BMI ≤23 kg/m^2^; ≤17.3 kg for BMI 23.1-26 kg/m^2^; ≤18 kg for BMI 26.1-29 kg/m^2^; or ≤21 kg for BMI >29 kg/m^2^; 0: Others
4	Slow gait speed: Participants were asked “How would you describe your usual walking pace?”	1: “Slow pace”; 0: Others
5	Low physical activity: Participants were asked “In the last 4 weeks, did you spend any time doing light DIY^a^ activity, heavy DIY activity, or strenuous sports?”	1: “None or light activity with a frequency of once per week or less”; 0: Others

^a^DIY: do it yourself.

### Covariates

Baseline data on age, sex (female or male), ethnicity (White, mixed race, South Asian, Black, Chinese, or other background), educational level (high, intermediate, or low), occupational status (working, retired, or other), alcohol consumption (never or special occasions only, 1 to 3 times per month, 1 to 4 times per week, or daily or almost daily), smoking status (never, previous smoker, or current smoker), healthy diet (yes or no), and family history of disease (including diabetes, CVD, dementia, and depression) were collected through a questionnaire interview. The Townsend deprivation index (TDI) was calculated based on areas before participants were recruited in the UKB. BMI was calculated as measured weight/height^2^ (kg/m^2^).

### Statistical Analyses

Baseline characteristics of the complete analyzed sample and by frailty status are presented as median (IQRs) and number (percentage) for continuous variables and categorical variables, respectively. Kruskal-Wallis tests and chi-square tests were used to compare the differences in characteristics by frailty status.

To evaluate the associations between frailty status (nonfrail, prefrail, and frail) and adverse outcomes, Cox proportional hazards regression models were performed. The Schoenfeld residuals test was used to verify the proportional hazard assumption, and no significant violation was found. We calculated hazard ratios (HRs) and corresponding 95% CIs using 2 models. Model 1 was adjusted for age and sex. Model 2 was further adjusted for ethnicity, educational level, occupational status, TDI, alcohol consumption, smoking status, healthy diet, BMI, and family history of disease based on Model 1. Additionally, we calculated HRs (95% CIs) for adverse outcomes per 1-point increase in FP score.

Several sensitivity analyses were conducted to confirm the robustness of the results. First, we compared the characteristics of included and excluded study participants. Second, to minimize the influence of reverse causality, we repeated the main analyses after excluding those without 2 years of follow-up. Third, to reduce the influence of poor health on frailty status, we repeated the main analyses after excluding participants with poor self-rated health status at baseline. Fourth, to account for the influence of missing data on results, we performed multiple imputations by chained equations [29] for missing values and repeated the primary analyses. Finally, we validated the associations between frailty and adverse outcomes among adults with T2DM. For adults with T2DM, HbA_1c_ level (≥7.0% [≥53 mmol/mol] or <7.0% [<53 mmol/mol]), diabetes medication use (oral antidiabetes drug only, insulin, or neither), diabetes duration (in years), and prevalent diabetes-related microvascular disease (except for incident diabetes-related microvascular disease) were also included in Model 2.

We used SAS version 9.4 (SAS Institute) and R version 3.6.3 (2020-02-29) to conduct all statistical analyses. To account for multiple testing, we used Bonferroni correction in all analyses (*P*<.006).

## Results

### Baseline Characteristics

Among the 38,950 participants with prediabetes, the median age was 58.6 (IQR 53.1-62.0) years, and the majority were women (21,155/38,950, 54.3%) and White (34,705/38,950, 89.1%; Table 2). The prevalences of prefrailty and frailty were 49.1% (19,122/38,950) and 5.9% (2289/38,950), respectively. Prefrail and frail adults were more likely to be women, have a lower educational level, and have a higher level of TDI and BMI, compared with the nonfrail adults. Table 2 shows the detailed baseline characteristics by frailty status.

**Table 2:** Baseline characteristics of study participants with prediabetes by frailty status.

Variables		Total (n=38,950)	Nonfrail (n=17,539)	Prefrail (n=19,122)	Frail (n=2289)	*P* value^a^
Age (years), median (IQR)		58.6 (53.1 to 62.0)	59.0 (53.7 to 62.1)	58.3 (52.7 to 61.8)	58.3 (52.9 to 61.7)	<.001
**Gender, n (%)**						<.001
	Female	21,155 (54.3)	8928 (50.9)	10,771 (56.3)	1456 (63.6)	
	Male	17,795 (45.7)	8611 (49.1)	8351 (43.7)	833 (36.4)	
**Ethnicity, n (%)**						<.001
	White	34,705 (89.1)	16,075 (91.7)	16,719 (87.4)	1911 (83.5)	
	Mixed	339 (0.9)	137 (0.8)	180 (0.9)	22 (1.0)	
	South Asian	1558 (4.0)	449 (2.6)	943 (4.9)	166 (7.3)	
	Black	1474 (3.8)	563 (3.2)	796 (4.2)	115 (5.0)	
	Chinese	261 (0.7)	104 (0.6)	139 (0.7)	18 (0.8)	
	Other background	613 (1.6)	211 (1.2)	345 (1.8)	57 (2.5)	
**Educational level^b^, n (%)**						<.001
	High	11,198 (28.7)	5647 (32.2)	5156 (27.0)	395 (17.3)	
	Intermediate	12,464 (32.0)	5728 (32.7)	6165 (32.2)	571 (24.9)	
	Low	15,288 (39.3)	6164 (35.1)	7801 (40.8)	1323 (57.8)	
**Occupational status, n (%)**						<.001
	Working	23,793 (61.1)	11,059 (63.1)	11,892 (62.2)	842 (36.8)	
	Retired	10,407 (26.7)	5095 (29.0)	4710 (24.6)	602 (26.3)	
	Other	4750 (12.2)	1385 (7.9)	2520 (13.2)	845 (36.9)	
Townsend deprivation index, median (IQR)		–1.7 (–3.5 to 1.2)	–2.2 (–3.7 to 0.3)	–1.4 (–3.2 to 1.6)	0.5 (–2.3 to 3.6)	<.001
BMI (kg/m^2^), median (IQR)		28.5 (25.4 to 32.1)	27.5 (24.8 to 30.8)	29.2 (25.9 to 32.9)	31.6 (27.8 to 36.4)	<.001
**Smoking status, n (%)**						<.001
	Never	19,301 (49.6)	8963 (51.1)	9366 (49.0)	972 (42.5)	
	Previous	12,788 (32.8)	5929 (33.8)	6137 (32.1)	722 (31.5)	
	Current	6861 (17.6)	2647 (15.1)	3619 (18.9)	595 (26.0)	
**Alcohol consumption, n (%)**						<.001
	Never or special occasions only	9939 (25.5)	3308 (18.9)	5551 (29.0)	1080 (47.2)	
	1 to 3 times per month	4919 (12.6)	2045 (11.7)	2587 (13.5)	287 (12.5)	
	1 to 4 times per week	17,545 (45.0)	8674 (49.5)	8176 (42.8)	695 (30.4)	
	Daily or almost daily	6547 (16.8)	3512 (20.0)	2808 (14.7)	227 (9.9)	
**Healthy diet, n (%)**						<.001
	No	9146 (23.5)	3444 (19.6)	4930 (25.8)	772 (33.7)	
	Yes	29,804 (76.5)	14,095 (80.4)	14,192 (74.2)	1517 (66.3)	
Glycated hemoglobin (mmol/mol), median (IQR)		40.4 (39.6 to 42.0)	40.3 (39.5 to 41.6)	40.5 (39.6 to 42.1)	40.9 (39.8 to 42.6)	<.001
**Prevalent diseases, n (%)**						
	Cardiovascular disease	3477 (8.9)	1157 (6.6)	1835 (9.6)	485 (21.2)	<.001
	Chronic kidney disease	180 (0.5)	55 (0.3)	88 (0.5)	37 (1.6)	<.001
	Eye disease	1303 (3.3)	520 (3.0)	646 (3.4)	137 (6.0)	<.001
	Dementia	14 (0.0)	4 (0.0)	7 (0.0)	3 (0.1)	.37
	Depression	2954 (7.6)	812 (4.6)	1636 (8.6)	506 (22.1)	<.001
**Family history, n (%)**						
	Diabetes mellitus	11,197 (28.7)	4716 (26.9)	5720 (29.9)	761 (33.2)	<.001
	Cardiovascular disease	23,633 (60.7)	10,448 (59.6)	11,711 (61.2)	1474 (64.4)	<.001
	Dementia	4733 (12.2)	2164 (12.3)	2283 (11.9)	286 (12.5)	.44
	Depression	5146 (13.2)	2087 (11.9)	2637 (13.8)	422 (18.4)	<.001

^a^Generated using chi-square and Kruskal-Wallis tests for categorical and continuous variables, respectively.

^b^Educational level was classified as high (college or university degree), intermediate (A/AS levels or equivalent, O levels/General Certificate of Secondary Education levels or equivalent), and low (none of the above).

### Frailty and Risks of Adverse Outcomes in Middle-aged Adults With Prediabetes

**Figure 2:**
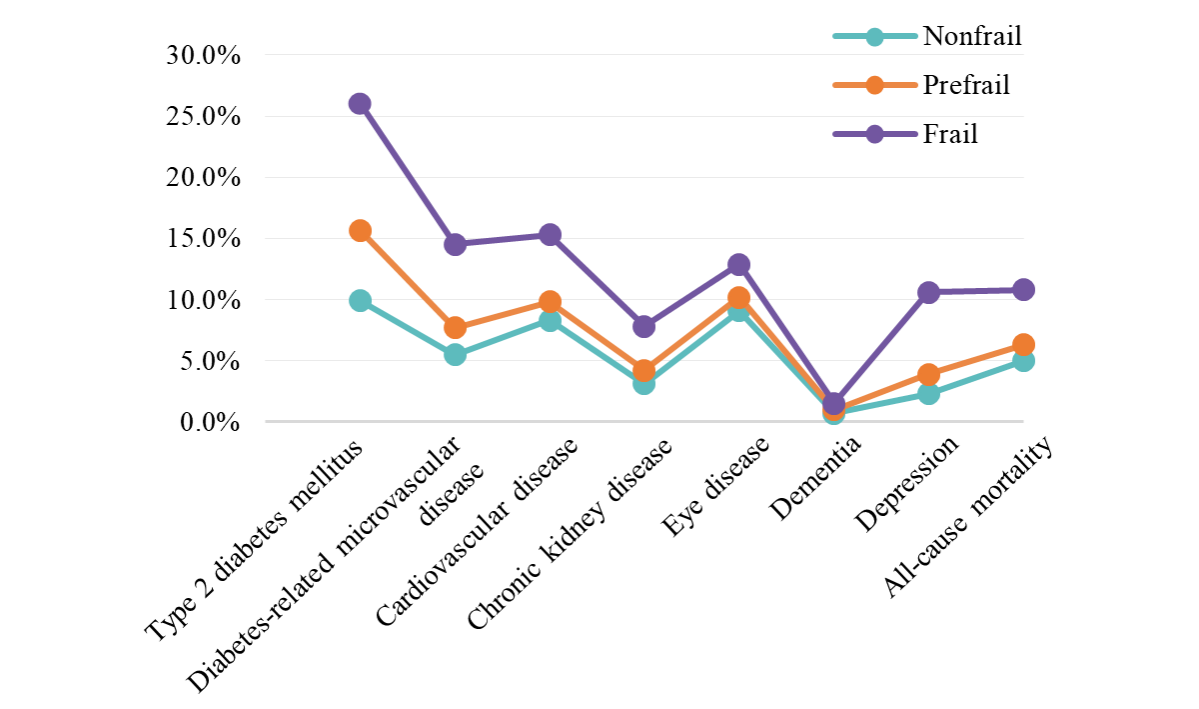
Age-adjusted incidence of adverse outcomes among UKB participants with prediabetes during 12 years of follow-up. UKB: UK Biobank.

During a median follow-up of 12 years, there were 5289 incident T2DM cases, 2657 incident diabetes-related microvascular disease cases, 3234 incident CVD cases, 1439 incident CKD cases, 3525 incident eye disease cases, 325 incident dementia cases, 1265 incident depression cases, and 2016 deaths. We found that frail participants developed more adverse outcomes than did their prefrail and nonfrail counterparts over the 12-year follow-up (Figure 2).

Table 3 shows the associations between frailty and the risks of multiple adverse outcomes in middle-aged adults with prediabetes. In the age- and sex-adjusted model, both prefrailty and frailty were associated with higher risks of all adverse outcomes (all *P* for trend <.001). After further adjusting for additional covariates, these associations remained statistically significant. When comparing prefrail participants with their nonfrail counterparts, the multivariable-adjusted HRs were 1.35 (95% CI 1.27-1.43) for T2DM, 1.29 (95% CI 1.18-1.40) for diabetes-related microvascular disease, 1.17 (95% CI 1.08-1.26) for CVD, 1.22 (95% CI 1.09-1.37) for CKD, 1.12 (95% CI 1.04-1.20) for eye disease, 1.57 (95% CI 1.23-2.01) for dementia, 1.48 (95% CI 1.30-1.68) for depression, and 1.25 (95% CI 1.14-1.38) for all-cause mortality. For frail participants, the multivariable-adjusted HRs were 1.73 (95% CI 1.55-1.92) for T2DM, 1.89 (95% CI 1.64-2.18) for diabetes-related microvascular disease, 1.66 (95% CI 1.44-1.91) for CVD, 1.76 (95% CI 1.45-2.13) for CKD, 1.31 (95% CI 1.14-1.51) for eye disease, 2.03 (95% CI 1.33-3.09) for dementia, 3.01 (95% CI 2.47-3.67) for depression, and 1.81 (95% CI 1.51-2.16) for all-cause mortality, compared with their nonfrail counterparts. Additionally, with each 1-point increase in FP score, the incidence risks of these adverse outcomes significantly increased by 10% to 42% (Model 2).

**Table 3:** Associations between frailty and adverse health outcomes among middle-aged adults with prediabetes.

Outcomes		Frailty status			*P* value for trend^a^	Hazard ratio (HR) per 1-point increase
		Nonfrail	Prefrail	Frail		
**Type 2 diabetes mellitus (n=38,950)**						
	Number of events/person-years	1724/207,929	2965/218,522	600/23,890	—^b^	—
	Model 1^c^, HR (95% CI)	Reference	1.70 (1.61-1.81)	3.37 (3.07-3.71)	<.001	1.45 (1.42-1.49)
	Model 2^d^, HR (95% CI)	Reference	1.35 (1.27-1.43)	1.73 (1.55-1.92)	<.001	1.19 (1.16-1.23)
**Diabetes-related microvascular disease (n=38,776)**						
	Number of events/person-years	926/212,240	1417/226,956	314/25,445	—	—
	Model 1^c^, HR (95% CI)	Reference	1.54 (1.42-1.67)	3.23 (2.84-3.68)	<.001	1.45 (1.40-1.50)
	Model 2^d^, HR (95% CI)	Reference	1.29 (1.18-1.40)	1.89 (1.64-2.18)	<.001	1.24 (1.19-1.29)
**Cardiovascular disease (n=35,473)**						
	Number of events/person-years	1314/195,009	1651/201,855	269/20,099	—	—
	Model 1^c^, HR (95% CI)	Reference	1.31 (1.22-1.41)	2.39 (2.09-2.72)	<.001	1.29 (1.25-1.34)
	Model 2^d^, HR (95% CI)	Reference	1.17 (1.08-1.26)	1.66 (1.44-1.91)	<.001	1.16 (1.12-1.21)
**Chronic kidney disease (n=38,770)**						
	Number of events/person-years	513/213,304	758/228,894	168/25,929	—	—
	Model 1^c^, HR (95% CI)	Reference	1.47 (1.31-1.64)	3.01 (2.53-3.58)	<.001	1.43 (1.36-1.50)
	Model 2^d^, HR (95% CI)	Reference	1.22 (1.09-1.37)	1.76 (1.45-2.13)	<.001	1.23 (1.16-1.30)
**Eye disease (n=37,647)**						
	Number of events/person-years	1470/202,556	1792/216,687	263/24,132	—	—
	Model 1^c^, HR (95% CI)	Reference	1.20 (1.12-1.29)	1.62 (1.42-1.85)	<.001	1.17 (1.13-1.21)
	Model 2^d^, HR (95% CI)	Reference	1.12 (1.04-1.20)	1.31 (1.14-1.51)	<.001	1.10 (1.06-1.14)
**Dementia (n=38,936)**						
	Number of events/person-years	111/215,549	181/232,270	33/26,881	—	—
	Model 1^c^, HR (95% CI)	Reference	1.69 (1.34-2.15)	2.87 (1.94-4.23)	<.001	1.41 (1.28-1.56)
	Model 2^d^, HR (95% CI)	Reference	1.57 (1.23-2.01)	2.03 (1.33-3.09)	<.001	1.29 (1.16-1.44)
**Depression (n=35,996)**						
	Number of events/person-years	387/204,125	687/209,605	191/19,994	—	—
	Model 1^c^, HR (95% CI)	Reference	1.71 (1.51-1.94)	4.97 (4.18-5.92)	<.001	1.63 (1.55-1.71)
	Model 2^d^, HR (95% CI)	Reference	1.48 (1.30-1.68)	3.01 (2.47-3.67)	<.001	1.42 (1.34-1.50)
**All-cause mortality (n=** **35,473** **)**						
	Number of events/person-years	783/195,777	1047/204,710	186/20,940	—	—
	Model 1^c^, HR (95% CI)	Reference	1.39 (1.27-1.53)	2.65 (2.26-3.12)	<.001	1.35 (1.29-1.41)
	Model 2^d^, HR (95% CI)	Reference	1.25 (1.14-1.38)	1.81 (1.51-2.16)	<.001	1.21 (1.16-1.27)

^a^Calculated to test linear trend using frailty status (3 categories) as a continuous variable.

^b^Not applicable.

^c^Model 1 was adjusted for age and sex.

^d^Model 2 was further adjusted for ethnicity, educational level, occupational status, Townsend deprivation index, alcohol consumption, smoking status, healthy diet, BMI, and family history of disease based on Model 1.

### Sensitivity Analyses

The differences in characteristics between included and excluded participants were observed. Those who were excluded were more likely to be older, women, non-White, and frail (Table S2 in Multimedia Appendix 1). Robust results were generally observed when excluding the participants with less than 2 years of follow-up (Table S3 in Multimedia Appendix 1), excluding the participants with poor self-rated health status at baseline (Table S4 in Multimedia Appendix 1), or imputing missing data on frailty and covariates (Table S5 in Multimedia Appendix 1). In addition, we confirmed that frailty was positively associated with the risks of diabetes-related microvascular disease, CVD, CKD, eye diseases, dementia, depression, and all-cause mortality in middle-aged adults with T2DM, and these associations were independent of factors related to diabetes severity at baseline (Table S6 in Multimedia Appendix 1).

## Discussion

### Principal Findings

In a large sample of UKB participants with prediabetes, we, for the first time, demonstrated that both prefrailty and frailty were associated with higher risks of multiple adverse outcomes, including T2DM, diabetes-related microvascular disease, CVD, CKD, eye disease, dementia, depression, and all-cause mortality. Our findings support the heterogeneity of prediabetes in middle-aged adulthood and suggest that assessing frailty status among middle-aged adults with prediabetes may help to identify those who were most at risk of subsequent adverse outcomes.

We observed a nearly twice higher prevalence of frailty among middle-aged adults with prediabetes (ie, 5.9%) in this study than that in general adults (ie, 3.3%) from the UKB as well [28]. Similarly, the prevalence of frailty among older adults with diabetes [30] is almost twice as high as that in those without diabetes (20.1% vs 12%) [31]. It seems that adults with glucose metabolism disorders are experiencing an accelerated aging process [32]. Multiple age-related metabolic disturbances are present in adults with prediabetes, including chronic inflammation, hyperglycemia, insulin resistance, and β-cell dysfunction [2,16], creating a pathophysiological environment that contributes to frailty. Given the sharp increase in frailty after the age of 65 years [33], our findings suggest that there is a need for early identification of frailty, an aging indicator, in this middle-aged population with prediabetes.

To the best of our knowledge, this study provided new evidence on the associations between frailty and higher risks of a series of adverse outcomes in middle-aged adults with prediabetes. A few studies on the relationship between frailty and adverse outcomes included middle-aged adults with diabetes as part of the study sample [19,20,34]. One prospective study of 998 African Americans aged 49 years to 65 years has shown that frail adults with diabetes had an increased risk of mortality [21]. Except for this study, only 1 study conducted in middle-aged and older adults with prediabetes found that frailty was associated with the progression of prediabetes to diabetes, as well as higher risks of CVD and all-cause mortality [23]. This large prospective study (n=38,950) showed that frailty was positively associated with higher risks of more outcomes including CKD, eye disease, and dementia in middle-aged adults with prediabetes.

This study draws attention to the accelerated aging process in adults with prediabetes, which may lead to rapid diabetes progression and contribute to the development of diabetes-related complications [32]. Nutritional and pharmacological anti-aging interventions have been revealed to help mitigate or reverse the accelerated aging process [35]. A recent review suggested that the most effective and easiest intervention strategy targeting frailty is to combine strength exercise and protein supplements in primary care [36]. Thus, our findings implicate that frailty assessment might help primary care providers identify the subpopulation at higher risk of adverse outcomes even in middle-aged adults with prediabetes in communities. It is worth noting that the application of technological solutions in assessing frailty is constantly expanding [37,38]. The major types of technologies include information and telecommunications technology–based platforms, smartphones, remote monitoring, and wearable sensors and devices [39]. For example, a frailty prediction model based on a points system and integrated into a mobile app for Android phones has been developed in the clinical setting, enabling professionals to identify frailty using clinical information and further improve decision-making [40]. With the aid of these technological tools, frailty screening becomes more convenient and flexible. Next, early preventive and interventive programs targeting frailty in adults with prediabetes are urgently needed. On the one hand, they may directly help reduce the occurrence of T2DM; on the other hand, they may indirectly help reduce diabetes-related burden. Meanwhile, pharmacologic interventions or other aggressive approaches to diabetes prevention are also encouraged [41,42]. Before formal implementation, considerably more research on the effectiveness and cost-effectiveness of interventional programs in this population is required.

### Strengths and Limitations

The major strengths of this study were the large sample of middle-aged adults with prediabetes, the long follow-up time, rich phenotype data, and linked hospital admissions records, enabling us to systematically evaluate the prospective associations between frailty and multiple adverse outcomes. There were several potential limitations. First, the UKB was not representative of the sampling population, and the majority of included adults were White. Also, there were differences in baseline characteristics between included and excluded participants. Thus, selection bias existed in this study, and the results may not be generalizable to populations from other countries. Second, transitions in frailty status may occur over time [43], and evidence has suggested that transitions in frailty status were associated with adverse outcomes [44]. However, repeated measurements of frailty were lacking; thus, we were unable to estimate the influence of frailty transitions on the subsequent adverse outcomes in this study. Future longitudinal studies incorporating data on frailty transition are needed. Third, multiple outcomes were considered in this study, and thus, type Ⅰ errors inevitably increased. To reduce the possibility of chance findings, we used Bonferroni correction. Finally, because of the observational study design, we could not draw a causal inference.

### Conclusion

In this prospective cohort study of middle-aged UKB participants with prediabetes, both prefrailty and frailty were significantly associated with increased risks of multiple adverse outcomes, including T2DM, diabetes-related microvascular disease, CVD, CKD, eye disease, dementia, depression, and all-cause mortality. The findings underscore the importance of frailty assessment in routine care for middle-aged adults with prediabetes. Detecting frailty at an early stage (ie, accelerated aging) and implementing timely targeted interventions may help to improve the allocation of health care resources and to reduce diabetes-related burden.

## Appendix

Multimedia Appendix 1 Supplementary tables.

## Abbreviations

ADA: American Diabetes Association
CKD: chronic kidney disease
CVD: cardiovascular disease
FP: frailty phenotype
HbA_1c_: glycated hemoglobin
HR: hazard ratio
ICD-9: International Statistical Classification of Diseases and Related Health Problems, 9th version
ICD-10: the International Statistical Classification of Diseases and Related Health Problems, 10th version
T2DM: type 2 diabetes mellitus
TDI: Townsend deprivation index
UKB: UK Biobank

## Data Availability

The data sets analyzed during this study are available at [45].
