# Supplementary material for: Associations Between Frailty and the Increased Risk of Adverse Outcomes Among 38,950 UK Biobank Participants With Prediabetes: Prospective Cohort Study
Source: JMIR Public Health Surveill. 2023 May 18;9:e45502. doi: 10.2196/45502 (PMC10236284; doi:10.2196/45502)
Supplement: Multimedia Appendix 1 [file publichealth_v9i1e45502_app1.docx]

Supplementary material (Online-only)

**List:**

**Table S1.** Codes used in the UK Biobank to define prevalent and incident diseases.

**Table S2.** Characteristics of the included and excluded study participants.

**Table S3.** Associations of frailty with adverse health outcomes among middle-aged adults with prediabetes after excluding the participants with less than 2 years of follow-up.

**Table S4.** Associations of frailty with adverse health outcomes among middle-aged adults with prediabetes after excluding the participants with poor self-rated health at baseline.

**Table S5.** Associations of frailty with adverse health outcomes among middle-aged adults with prediabetes after multiple imputations for missing data.

**Table S6.** Associations of frailty with adverse health outcomes among middle-aged adults with type 2 diabetes mellitus.

**Table S1.** Codes used in the UK Biobank to define prevalent and incident diseases.

| **Diseases** | **Self-reported**^a^ | **ICD-9** | **ICD-10** |
| --- | --- | --- | --- |
| Type 2 diabetes mellitus | 20002 (1223) | 41271 (25000, 25010, 25020, 25090) | 41270 (E11) |
| Diabetes-related microvascular disease | — | 41271 (2503, V420, 7910, 5845, 5846, 5847, 5848, 5849, 5859, 5869, V451, V560, V568, 2504, 2505, 3620, 3572) | 41270 (E112, E142, Z940, N083, R80, N391, N170, N171, N172, N178, N179, N180, N181, N182, N183, N184, N185, N188, N189, N19, Z992, Z490, Z491, Z492, E113, E143, H280, H360, E114, E144, G730, G990, G590, G632) |
| Cardiovascular disease | 20002 (1066, 1074, 1075, 1081, 1086, 1491, 1583) | 41271 (410, 411, 412, 413, 414, 42979, 430, 431, 432, 433, 434, 435, 436, 437, 438) | 41270 (I20, I21, I22, I23, I241, I25, I46, I60, I61, I63, I64) |
| Chronic kidney disease | 20002 (1427, 1192, 1193, 1194, 1519, 1520) | 41271 (7531, 585, 586) | 41270 (Q61, N18, N19) |
| Eye disease | 20002 (1278, 1277) | 41271 (3661, 3662, 3663, 3665, 3668, 3669, 3650, 3651, 3652, 3655, 3656, 3659) | 41270 (H25, H26, H40) |
| Dementia | 20002 (1263) | 41271 (2902, 2903, 2904, 2912, 2941, 3310, 3311, 3312, 3315) | 41270 (F00, F000, F001, F002, F009, G30, G300, G301, G308, G309, F01, F010, F011, F012, F013, F018, F019, I673, F020, G310, A810, F02, F021, F022, F023, F024, F028, F03, F051, F106, G311, G318) |
| Depression | 20002 (1286, 1531) | — | 41270 (F32-F34, F38-F39) |

^a^Self-reported codes were only used to define prevalent diseases. Eye disease includes cataract and glaucoma.

**Table 2.** Characteristics of the included and excluded study participants.^a^

| **Variables** | **Total (N=43,133)** | **Included (N=38,950)** | **Excluded (N=4183)** | ***P* value**^b^ |
| --- | --- | --- | --- | --- |
| Age (years), median (IQR) | 58.7 (53.2, 62.0) | 58.6 (53.1, 62.0) | 59.5 (53.9, 62.4) | <.001 |
| **Gender, n (%)** |  |  |  | <.001 |
| Female | 23,660 (54.9) | 21,155 (54.3) | 2505 (59.9) |  |
| Male | 19,473 (45.1) | 17,795 (45.7) | 1678 (40.1) |  |
| **Ethnicity, n (%)** |  |  |  | <.001 |
| White | 37,962 (88.6) | 34,705 (89.1) | 3257 (83.6) |  |
| Mixed | 381 (0.9) | 339 (0.9) | 42 (1.1) |  |
| South Asian | 1833 (4.3) | 1558 (4.0) | 275 (7.1) |  |
| Black | 1656 (3.9) | 1474 (3.8) | 182 (4.7) |  |
| Chinese | 294 (0.7) | 261 (0.7) | 33 (0.8) |  |
| Other background | 720 (1.7) | 613 (1.6) | 107 (2.7) |  |
| **Educational level**^c^**, n (%)** |  |  |  | .02 |
| High | 12,193 (28.7) | 11,198 (28.7) | 995 (28.0) |  |
| Intermediate | 13,544 (31.9) | 12,464 (32.0) | 1080 (30.4) |  |
| Low | 16,770 (39.5) | 15,288 (39.3) | 1482 (41.7) |  |
| **Occupational status, n (%)** |  |  |  | <.001 |
| Working | 25,646 (59.9) | 23,793 (61.1) | 1853 (48.2) |  |
| Retired | 11,708 (27.4) | 10,407 (26.7) | 1301 (33.8) |  |
| Other | 5440 (12.7) | 4750 (12.2) | 690 (18.0) |  |
| Townsend deprivation index, median (IQR) | -1.7 (-3.4, 1.3) | -1.7 (-3.5, 1.2) | -1.2 (-3.2, 2.1) | <.001 |
| BMI (kg/m^2^), median (IQR) | 28.5 (25.4, 32.1) | 28.5 (25.4, 32.1) | 28.4 (25.3, 32.0) | .17 |
| **Smoking status, n (%)** |  |  |  | .97 |
| Never | 21,234 (49.6) | 19,301 (49.6) | 1933 (49.7) |  |
| Previous | 14,057 (32.8) | 12,788 (32.8) | 1269 (32.6) |  |
| Current | 7548 (17.6) | 6861 (17.6) | 687 (17.7) |  |
| **Alcohol consumption, n (%)** |  |  |  | <.001 |
| Never or special occasions only | 11,285 (26.3) | 9939 (25.5) | 1346 (33.5) |  |
| 1 to 3 times per month | 5410 (12.6) | 4919 (12.6) | 491 (12.2) |  |
| 1 to 4 times per week | 19,208 (44.7) | 17,545 (45.0) | 1663 (41.4) |  |
| Daily or almost daily | 7067 (16.4) | 6547 (16.8) | 520 (12.9) |  |
| **Healthy diet, n (%)** |  |  |  | .40 |
| No | 10,108 (23.5) | 9146 (23.5) | 962 (24.1) |  |
| Yes | 32,838 (76.5) | 29,804 (76.5) | 3034 (75.9) |  |
| Glycated hemoglobin (mmol/mol), median (IQR) | 40.5 (39.6, 42.0) | 40.4 (39.6, 42.0) | 40.6 (39.6, 42.1) | <.001 |
| **Prevalent diseases, n (%)** |  |  |  |  |
| Cardiovascular disease | 3874 (9.0) | 3477 (8.9) | 397 (9.5) | .23 |
| Chronic kidney disease | 217 (0.5) | 180 (0.5) | 37 (0.9) | <.001 |
| Eye disease | 1463 (3.4) | 1303 (3.3) | 160 (3.8) | .10 |
| Dementia | 19 (0.0) | 14 (0.0) | 5 (0.1) | .01 |
| Depression | 3313 (7.7) | 2954 (7.6) | 359 (8.6) | .02 |
| **Family history, n (%)** |  |  |  |  |
| Diabetes mellitus | 12,257 (28.7) | 11,197 (28.7) | 1060 (28.7) | .99 |
| Cardiovascular disease | 25,818 (60.6) | 23,633 (60.7) | 2185 (59.2) | .09 |
| Dementia | 5139 (12.1) | 4733 (12.2) | 406 (11.0) | .04 |
| Depression | 5610 (13.2) | 5146 (13.2) | 464 (12.6) | .28 |
| **Frailty status, n (%)** |  |  |  |  |
| Nonfrail | 19,184 (44.5) | 17,539 (45.0) | 1645 (39.4) | <.001 |
| Prefrail | 21,304 (49.4) | 19,122 (49.1) | 2182 (52.3) |  |
| Frail | 2638 (6.1) | 2289 (5.9) | 349 (8.4) |  |

^a^Data are expressed as numbers and percentages for categorical variables, and medians and inter-quartile range (IQR) for continuous variables. There were missing data on ethnicity (n=287), educational level (n=626), occupational status (n=339), Townsend deprivation index (n=71), BMI (n=226), smoking status (n=294), alcohol consumption (n=163), healthy diet (n=187), family history of diabetes mellitus, cardiovascular disease, dementia, or depression (n=495), and frailty (n=7).

^b^Generated using chi-square and Kruskal-Wallis test for categorical and continuous variables, respectively.

^c^The educational level was classified as high (college or university degree), intermediate (A/AS levels or equivalent, O levels/General Certificate of Secondary Education levels or equivalent), and low (none of the above).

**Table S3.** Associations of frailty with adverse health outcomes among middle-aged adults with prediabetes after excluding the participants with less than 2 years of follow-up.

| **Outcomes** | **Frailty status** | | | ***P* value for trend**^a^ | **Hazard ratio (HR) per 1-point increase** |
| --- | --- | --- | --- | --- | --- |
|  | **Nonfrail** | **Prefrail** | **Frail** |  |  |
| **Type 2 diabetes mellitus (N=38,565)** | | | |  |  |
| No. of events/No. of participants | 1658/17,419 | 2849/18,916 | 567/2230 | —^b^ | — |
| Model 1^c^, HR (95% CI) | Ref. | 1.70 (1.60, 1.81) | 3.34 (3.03, 3.67) | <.001 | 1.45 (1.41, 1.48) |
| Model 2^d^, HR (95% CI) | Ref. | 1.35 (1.27, 1.44) | 1.72 (1.54, 1.92) | <.001 | 1.19 (1.16, 1.22) |
|  |  |  |  |  |  |
| **Diabetes-related microvascular disease (N=38,394)** | |  |  |  |  |
| No. of events/No. of participants | 909/17,365 | 1387/18,838 | 298/2191 | — | — |
| Model 1^c^, HR (95% CI) | Ref. | 1.54 (1.42, 1.67) | 3.16 (2.77, 3.61) | <.001 | 1.44 (1.39, 1.49) |
| Model 2^d^, HR (95% CI) | Ref. | 1.29 (1.18, 1.41) | 1.86 (1.60, 2.15) | <.001 | 1.24 (1.19, 1.29) |
|  |  |  |  |  |  |
| **Cardiovascular disease (N=34,942)** |  |  |  |  |  |
| No. of events/No. of participants | 1178/16,203 | 1429/16,995 | 227/1744 | — | — |
| Model 1^c^, HR (95% CI) | Ref. | 1.27 (1.17, 1.37) | 2.27 (1.96, 2.61) | <.001 | 1.26 (1.22, 1.31) |
| Model 2^d^, HR (95% CI) | Ref. | 1.13 (1.04, 1.22) | 1.58 (1.36, 1.85) | <.001 | 1.14 (1.09, 1.19) |
|  |  |  |  |  |  |
| **Chronic kidney disease (N=38,557)** |  |  |  |  |  |
| No. of events/No. of participants | 504/17,421 | 733/18,918 | 160/2218 | — | — |
| Model 1^c^, HR (95% CI) | Ref. | 1.45 (1.29, 1.62) | 2.92 (2.44, 3.49) | <.001 | 1.42 (1.35, 1.49) |
| Model 2^d^, HR (95% CI) | Ref. | 1.20 (1.07, 1.35) | 1.69 (1.38, 2.06) | <.001 | 1.21 (1.15, 1.28) |
|  |  |  |  |  |  |
| **Eye disease (N=37,250)** |  |  |  |  |  |
| No. of events/No. of participants | 1378/16,872 | 1674/18,271 | 244/2107 | — | — |
| Model 1^c^, HR (95% CI) | Ref. | 1.20 (1.12, 1.29) | 1.61 (1.40, 1.85) | <.001 | 1.16 (1.12, 1.21) |
| Model 2^d^, HR (95% CI) | Ref. | 1.12 (1.04, 1.20) | 1.31 (1.13, 1.51) | <.001 | 1.10 (1.06, 1.14) |
|  |  |  |  |  |  |
| **Dementia (N=38,752)** |  |  |  |  |  |
| No. of events/No. of participants | 109/17,477 | 175/19,017 | 33/2258 | — | — |
| Model 1^c^, HR (95% CI) | Ref. | 1.67 (1.32, 2.13) | 2.94 (1.99, 4.33) | <.001 | 1.42 (1.29, 1.57) |
| Model 2^d^, HR (95% CI) | Ref. | 1.55 (1.21, 1.98) | 2.07 (1.35, 3.16) | <.001 | 1.30 (1.16, 1.45) |
|  |  |  |  |  |  |
| **Depression (N=35,742)** |  |  |  |  |  |
| No. of events/No. of participants | 357/16,648 | 639/17,355 | 169/1739 | — | — |
| Model 1^c^, HR (95% CI) | Ref. | 1.73 (1.52, 1.97) | 4.81 (4.00, 5.78) | <.001 | 2.07 (1.88, 2.29) |
| Model 2^d^, HR (95% CI) | Ref. | 1.51 (1.32, 1.73) | 2.99 (2.43, 3.68) | <.001 | 1.67 (1.50, 1.86) |
|  |  |  |  |  |  |
| **All-cause mortality (N=35,333)** |  |  |  |  |  |
| No. of events/No. of participants | 739/16,336 | 982/17,212 | 169/1785 | — | — |
| Model 1^c^, HR (95% CI) | Ref. | 1.38 (1.26, 1.52) | 2.56 (2.16, 3.02) | <.001 | 1.33 (1.27, 1.39) |
| Model 2^d^, HR (95% CI) | Ref. | 1.24 (1.12, 1.37) | 1.73 (1.44, 2.08) | <.001 | 1.19 (1.13, 1.25) |

^a^Calculated to test linear trend using frailty status (three-categorical) as a continuous variable.

^b^Not applicable.

^c^Model 1 was adjusted for age and sex.

^d^Model 2 was further adjusted for ethnicity, educational level, occupational status, Townsend deprivation index, alcohol consumption, smoking status, healthy diet, BMI, and family history of disease based on Model 1.

**Table S4.** Associations of frailty with adverse health outcomes among middle-aged adults with prediabetes after excluding the participants with poor self-rated health at baseline.

| **Outcomes** | **Frailty status** | | | ***P* value for trend**^a^ | **Hazard ratio (HR) per 1-point increase** |
| --- | --- | --- | --- | --- | --- |
|  | **Nonfrail** | **Prefrail** | **Frail** |  |  |
| **Type 2 diabetes mellitus (N=36,257)** | | | |  |  |
| No. of events/No. of participants | 1665/17,235 | 2576/17,621 | 317/1401 | —^b^ | — |
| Model 1^c^, HR (95% CI) | Ref. | 1.63 (1.53, 1.73) | 2.94 (2.60, 3.32) | <.001 | 1.42 (1.38, 1.46) |
| Model 2^d^, HR (95% CI) | Ref. | 1.31 (1.23, 1.39) | 1.56 (1.37, 1.79) | <.001 | 1.17 (1.13, 1.21) |
|  |  |  |  |  |  |
| **Diabetes-related microvascular disease (N=36,129)** | |  |  |  |  |
| No. of events/No. of participants | 882/17,182 | 1196/175,640 | 169/1383 | — | — |
| Model 1^c^, HR (95% CI) | Ref. | 1.45 (1.32, 1.58) | 2.90 (2.45, 3.42) | <.001 | 1.38 (1.32, 1.45) |
| Model 2^d^, HR (95% CI) | Ref. | 1.23 (1.13, 1.35) | 1.85 (1.55, 2.21) | <.001 | 1.21 (1.15, 1.27) |
|  |  |  |  |  |  |
| **Cardiovascular disease (N=33,507)** |  |  |  |  |  |
| No. of events/No. of participants | 1279/16,142 | 1462/16,163 | 159/1202 | — | — |
| Model 1^c^, HR (95% CI) | Ref. | 1.25 (1.16, 1.35) | 2.14 (1.81, 2.52) | <.001 | 1.24 (1.19, 1.29) |
| Model 2^d^, HR (95% CI) | Ref. | 1.13 (1.04, 1.22) | 1.60 (1.34, 1.90) | <.001 | 1.13 (1.08, 1.18) |
|  |  |  |  |  |  |
| **Chronic kidney disease (N=36,124)** |  |  |  |  |  |
| No. of events/No. of participants | 484/17,182 | 636/17,556 | 94/1386 | — | — |
| Model 1^c^, HR (95% CI) | Ref. | 1.38 (1.23, 1.56) | 2.78 (2.23, 3.48) | <.001 | 1.36 (1.28, 1.45) |
| Model 2^d^, HR (95% CI) | Ref. | 1.18 (1.05, 1.34) | 1.79 (1.41, 2.28) | <.001 | 1.20 (1.12, 1.28) |
|  |  |  |  |  |  |
| **Eye disease (N=35,105)** |  |  |  |  |  |
| No. of events/No. of participants | 1431/16,620 | 1605/16,804 | 146/1258 | — | — |
| Model 1^c^, HR (95% CI) | Ref. | 1.17 (1.09, 1.26) | 1.42 (1.20, 1.68) | <.001 | 1.13 (1.09, 1.18) |
| Model 2^d^, HR (95% CI) | Ref. | 1.10 (1.02, 1.18) | 1.18 (0.99, 1.40) | .005 | 1.07 (1.03, 1.11) |
|  |  |  |  |  |  |
| **Dementia (N=36,249)** |  |  |  |  |  |
| No. of events/No. of participants | 106/17,232 | 150/17,617 | 14/1400 | — | — |
| Model 1^c^, HR (95% CI) | Ref. | 1.56 (1.21, 2.00) | 2.01 (1.15, 3.52) | <.001 | 1.29 (1.14, 1.46) |
| Model 2^d^, HR (95% CI) | Ref. | 1.49 (1.15, 1.93) | 1.73 (0.97, 3.07) | .001 | 1.24 (1.09, 1.42) |
|  |  |  |  |  |  |
| **Depression (N=33,939)** |  |  |  |  |  |
| No. of events/No. of participants | 365/16,457 | 569/16,291 | 112/1191 | — | — |
| Model 1^c^, HR (95% CI) | Ref. | 1.58 (1.39, 1.80) | 4.47 (3.61, 5.53) | <.001 | 1.89 (1.69, 2.12) |
| Model 2^d^, HR (95% CI) | Ref. | 1.40 (1.22, 1.60) | 3.10 (2.46, 3.91) | <.001 | 1.61 (1.43, 1.81) |
|  |  |  |  |  |  |
| **All-cause mortality (N=33,507)** |  |  |  |  |  |
| No. of events/No. of participants | 763/16,142 | 916/16,163 | 107/1202 | — | — |
| Model 1^c^, HR (95% CI) | Ref. | 1.31 (1.19, 1.44) | 2.27 (1.85, 2.78) | <.001 | 1.28 (1.21, 1.35) |
| Model 2^d^, HR (95% CI) | Ref. | 1.20 (1.08, 1.32) | 1.74 (1.41, 2.16) | <.001 | 1.19 (1.12, 1.25) |

^a^Calculated to test linear trend using frailty status (three-categorical) as a continuous variable.

^b^Not applicable.

^c^Model 1 was adjusted for age and sex.

^d^Model 2 was further adjusted for ethnicity, educational level, occupational status, Townsend deprivation index, alcohol consumption, smoking status, healthy diet, BMI, and family history of disease based on Model 1.

**Table S5.** Associations of frailty with adverse health outcomes among middle-aged adults with prediabetes after multiple imputations for missing data.

| **Outcomes** | **Frailty status** | | | ***P* value for trend**^a^ | **Hazard ratio (HR) per 1-point increase** |
| --- | --- | --- | --- | --- | --- |
|  | **Nonfrail** | **Prefrail** | **Frail** |  |  |
| **Type 2 diabetes mellitus (N=40,747)** |  |  |  |  |  |
| No. of events/Person-years | 1807/215,585 | 3151/229,165 | 634/25,443 | —^b^ | — |
| Model 1^c^, HR (95% CI) | Ref. | 1.71 (1.61, 1.81) | 3.30 (3.01, 3.61) | <.001 | 1.45 (1.41, 1.48) |
| Model 2^d^, HR (95% CI) | Ref. | 1.35 (1.27, 1.43) | 1.71 (1.54, 1.90) | <.001 | 1.19 (1.16, 1.22) |
|  |  |  |  |  |  |
| **Diabetes-related microvascular disease (N=40,566)** | | | |  |  |
| No. of events/Person-years | 967/220,155 | 1513/238,132 | 334/27,092 | — | — |
| Model 1^c^, HR (95% CI) | Ref. | 1.56 (1.44, 1.69) | 3.21 (2.83, 3.64) | <.001 | 1.45 (1.40, 1.50) |
| Model 2^d^, HR (95% CI) | Ref. | 1.30 (1.20, 1.41) | 1.88 (1.64, 2.16) | <.001 | 1.24 (1.19, 1.29) |
|  |  |  |  |  |  |
| **Cardiovascular disease (N=37,077)** |  |  |  |  |  |
| No. of events/Person-years | 1360/202,183 | 1,750/211,681 | 1642/21,421 | — | — |
| Model 1^c^, HR (95% CI) | Ref. | 1.33 (1.24, 1.43) | 2.35 (2.07, 2.68) | <.001 | 1.29 (1.25, 1.34) |
| Model 2^d^, HR (95% CI) | Ref. | 1.19 (1.10, 1.28) | 1.67 (1.45, 1.92) | <.001 | 1.17 (1.13, 1.22) |
|  |  |  |  |  |  |
| **Chronic kidney disease (N=40,558)** |  |  |  |  |  |
| No. of events/Person-years | 530/221,305 | 813/240,210 | 175/27,609 | — | — |
| Model 1^c^, HR (95% CI) | Ref. | 1.51 (1.35, 1.68) | 2.95 (2.49, 3.51) | <.001 | 1.43 (1.36, 1.50) |
| Model 2^d^, HR (95% CI) | Ref. | 1.26 (1.12, 1.40) | 1.74 (1.44, 2.10) | <.001 | 1.23 (1.17, 1.30) |
|  |  |  |  |  |  |
| **Eye disease (N=39,372)** |  |  |  |  |  |
| No. of events/Person-years | 1523/210,013 | 1891/227,360 | 283/25,682 | — | — |
| Model 1^c^, HR (95% CI) | Ref. | 1.21 (1.13, 1.29) | 1.64 (1.45, 1.87) | <.001 | 1.17 (1.13, 1.21) |
| Model 2^d^, HR (95% CI) | Ref. | 1.12 (1.05, 1.20) | 1.33 (1.16, 1.53) | <.001 | 1.10 (1.06, 1.14) |
|  |  |  |  |  |  |
| **Dementia (N=40,730)** |  |  |  |  |  |
| No. of events/Person-years | 118/223,606 | 196/243,793 | 36/28,606 | — | — |
| Model 1^c^, HR (95% CI) | Ref. | 1.71 (1.36, 2.14) | 2.87 (1.98, 4.17) | <.001 | 1.42 (1.29, 1.56) |
| Model 2^d^, HR (95% CI) | Ref. | 1.56 (1.23, 1.97) | 1.94 (1.29, 2.92) | <.001 | 1.28 (1.15, 1.42) |
|  |  |  |  |  |  |
| **Depression (N=37,639)** |  |  |  |  |  |
| No. of events/Person-years | 408/211,606 | 744/219,903 | 204/21,362 | — | — |
| Model 1^c^, HR (95% CI) | Ref. | 1.74 (1.54, 1.96) | 4.90 (4.14, 5.80) | <.001 | 1.63 (1.55, 1.70) |
| Model 2^d^, HR (95% CI) | Ref. | 1.50 (1.32, 1.70) | 2.97 (2.45, 3.59) | <.001 | 1.41 (1.34, 1.49) |
|  |  |  |  |  |  |
| **All-cause mortality (N=37,077)** |  |  |  |  |  |
| No. of events/Person-years | 824/202,959 | 1118/214,767 | 205/22,289 | — | — |
| Model 1^c^, HR (95% CI) | Ref. | 1.39 (1.27, 1.53) | 2.70 (2.31, 3.15) | <.001 | 1.36 (1.30, 1.42) |
| Model 2^d^, HR (95% CI) | Ref. | 1.25 (1.14, 1.38) | 1.83 (1.55, 2.17) | <.001 | 1.22 (1.17, 1.28) |

^a^Calculated to test linear trend using frailty status (three-categorical) as a continuous variable.

^b^Not applicable.

^c^Model 1 was adjusted for age and sex.

^d^Model 2 was further adjusted for ethnicity, educational level, occupational status, Townsend deprivation index, alcohol consumption, smoking status, healthy diet, BMI, and family history of disease based on Model 1.

**Table S6.** Associations of frailty with adverse health outcomes among middle-aged adults with type 2 diabetes mellitus.

| **Outcomes** | **Frailty status** | | | ***P* value for trend**^a^ | **Hazard ratio (HR) per 1-point increase** |
| --- | --- | --- | --- | --- | --- |
|  | **Nonfrail** | **Prefrail** | **Frail** |  |  |
| **Diabetes-related microvascular disease (N=13,650)** | | | |  |  |
| No. of events/Person-years | 685/44,628 | 1,670/87,150 | 592/19,060 | —^b^ | — |
| Model 1^c^, HR (95% CI) | Ref. | 1.31 (1.20, 1.43) | 2.25 (2.01, 2.51) | <.001 | 1.28 (1.25, 1.33) |
| Model 2^d^, HR (95% CI) | Ref. | 1.16 (1.06, 1.27) | 1.55 (1.37, 1.75) | <.001 | 1.16 (1.12, 1.20) |
|  |  |  |  |  |  |
| **Cardiovascular disease (N=11,597)** |  |  |  |  |  |
| No. of events/Person-years | 467/39,703 | 1,019/75,934 | 277/14,814 | — | — |
| Model 1^c^, HR (95% CI) | Ref. | 1.21 (1.09, 1.35) | 1.83 (1.58, 2.13) | <.001 | 1.21 (1.16, 1.26) |
| Model 2^d^, HR (95% CI) | Ref. | 1.08 (0.97, 1.21) | 1.30 (1.10, 1.53) | .004 | 1.09 (1.04, 1.14) |
|  |  |  |  |  |  |
| **Chronic kidney disease (N=13,863)** |  |  |  |  |  |
| No. of events/Person-years | 286/46,800 | 759/92,664 | 331/20,883 | — | — |
| Model 1^c^, HR (95% CI) | Ref. | 1.42 (1.24, 1.63) | 2.88 (2.46, 3.38) | <.001 | 1.37 (1.31, 1.44) |
| Model 2^d^, HR (95% CI) | Ref. | 1.17 (1.02, 1.35) | 1.70 (1.43, 2.04) | <.001 | 1.18 (1.12, 1.24) |
|  |  |  |  |  |  |
| **Eye disease (N=13,313)** |  |  |  |  |  |
| No. of events/Person-years | 491/44,164 | 1,070/86,783 | 350/19,323 | — | — |
| Model 1^c^, HR (95% CI) | Ref. | 1.15 (1.03, 1.28) | 1.68 (1.46, 1.93) | <.001 | 1.17 (1.12, 1.21) |
| Model 2^d^, HR (95% CI) | Ref. | 1.07 (0.96, 1.19) | 1.39 (1.20, 1.62) | <.001 | 1.11 (1.06, 1.16) |
|  |  |  |  |  |  |
| **Dementia (N=14,019)** |  |  |  |  |  |
| No. of events/Person-years | 44/47,979 | 141/96,240 | 64/22,587 | — | — |
| Model 1^c^, HR (95% CI) | Ref. | 1.77 (1.26, 2.48) | 3.60 (2.45, 5.29) | <.001 | 1.43 (1.29, 1.57) |
| Model 2^d^, HR (95% CI) | Ref. | 1.68 (1.19, 2.37) | 2.87 (1.87, 4.40) | <.001 | 1.34 (1.20, 1.50) |
|  |  |  |  |  |  |
| **Depression (N=12,750)** |  |  |  |  |  |
| No. of events/Person-years | 118/45,380 | 420/86,207 | 174/17,398 | — | — |
| Model 1^c^, HR (95% CI) | Ref. | 1.84 (1.50, 2.25) | 3.78 (2.99, 4.78) | <.001 | 1.96 (1.74, 2.21) |
| Model 2^d^, HR (95% CI) | Ref. | 1.61 (1.30, 1.98) | 2.43 (1.87, 3.15) | <.001 | 1.28 (1.19, 1.37) |
|  |  |  |  |  |  |
| **All-cause mortality (N=11,597)** |  |  |  |  |  |
| No. of events/Person-years | 263/40,984 | 614/79,019 | 204/15,770 | — | — |
| Model 1^c^, HR (95% CI) | Ref. | 1.32 (1.14, 1.52) | 2.45 (2.04, 2.95) | <.001 | 1.33 (1.27, 1.41) |
| Model 2^d^, HR (95% CI) | Ref. | 1.15 (0.99, 1.34) | 1.54 (1.25, 1.89) | <.001 | 1.16 (1.10, 1.23) |

^a^Calculated to test linear trend using frailty status (three-categorical) as a continuous variable.

^b^Not applicable.

^c^Model 1 was adjusted for age and sex.

^d^Model 2 was further adjusted for ethnicity, educational level, occupational status, Townsend deprivation index, alcohol consumption, smoking status, healthy diet, BMI, family history of disease, glycated hemoglobin level (≥7.0% [≥53 mmol/mol], or <7.0% [<53 mmol/mol]), diabetes medication use (oral antidiabetes drug only, insulin, or neither), diabetes duration (in years), and diabetes-related microvascular disease (except for incident diabetes-related microvascular disease) based on Model 1.
